# Arachidonic Acid Metabolites in Cardiovascular and Metabolic Diseases

**DOI:** 10.3390/ijms19113285

**Published:** 2018-10-23

**Authors:** Thomas Sonnweber, Alex Pizzini, Manfred Nairz, Günter Weiss, Ivan Tancevski

**Affiliations:** Department of Internal Medicine II, Medical University Innsbruck, Innsbruck 6020, Austria; Thomas.Sonnweber@i-med.ac.at (T.S.); Alex.Pizzini@i-med.ac.at (A.P.); Manfred.Nairz@i-med.ac.at (M.N.); guenter.weiss@i-med.ac.at (G.W.)

**Keywords:** arachidonic acid, eicosanoids, metabolic disease, obesity, nonalcoholic fatty liver disease, diabetes, atherosclerosis, cardiovascular disease, cholesterol

## Abstract

Lipid and immune pathways are crucial in the pathophysiology of metabolic and cardiovascular disease. Arachidonic acid (AA) and its derivatives link nutrient metabolism to immunity and inflammation, thus holding a key role in the emergence and progression of frequent diseases such as obesity, diabetes, non-alcoholic fatty liver disease, and cardiovascular disease. We herein present a synopsis of AA metabolism in human health, tissue homeostasis, and immunity, and explore the role of the AA metabolome in diverse pathophysiological conditions and diseases.

## 1. Introduction

An overlapping incidence of obesity and various metabolic, as well as cardiovascular diseases, has been recognized for a long time [1,2]. Despite improvements in the treatment and the reduction of mortality from cardiovascular diseases within the last years, the tremendous increase in the prevalence of obesity and its associated comorbidities threatens to subvert these advances [3]. By 2010 the prevalence of obesity, as defined by a body mass index (BMI) above 30, has spiked to 36 percent of female and male adults in the United States [3], and the World Health Organization (WHO) estimated that, in 2016, over 1.9 billion adults and 340 million children and adolescents were overweight or obese worldwide [4]. These alarming numbers indicate that obesity nearly tripled within the last 40 years, with increasing numbers of obesity-associated diseases that are considered a major challenge of modern medicine. Over- or malnutrition, especially high fat and carbohydrate intake, lack of exercise and sedentary lifestyles are the main causes for weight gain and obesity. The latter is associated with a plethora of metabolic and cardiovascular diseases, including diabetes mellitus (DM), non-alcoholic fatty liver disease (NAFLD), non-alcoholic steatohepatitis (NASH), atherosclerosis, myocardial infarction and stroke, which have a major impact on mortality worldwide [5,6,7].

Early studies defined obesity and its related morbidities as simple lipid-storage diseases [8,9,10]. Still, the understanding of the pathophysiology involved in obesity-associated diseases has vastly increased within the last decades, pointing towards shared features of altered nutrient metabolism, and chronic inflammation to be key components in disease development [11,12,13,14,15]. In this context, arachidonic acid (AA) metabolism provides an interesting link between lipid metabolism and immunity and was implicated in the emergence and course of cardiovascular and metabolic diseases [16]. We herein review the current knowledge of the regulation of AA metabolism and its impact on human health and disease focusing on recent advances in the understanding of AA metabolism in obesity, DM, NAFLD/NASH, atherosclerosis, and cardiovascular disease.

## 2. AA Metabolism

In the context of nutrition and medicine, the term AA typically refers to all-*cis*-5,8,11,14-eicosatetraenoic acid, an omega-6 polyunsaturated fatty acid (PUFA), which consists of twenty carbon chains and four *cis* double bonds. PUFAs are abundantly present in phospholipids of human cell membranes, and in lipid droplets (LDs) in immune cells [17,18,19,20]. According to the twenty carbon units in length, AA and its derivatives, as well as various omega-3 PUFAs, are also termed eicosanoids. The highest concentrations of AA are found in the brain, muscle, liver, spleen, and retina, whereas concentrations of free AA in the circulation are typically very low due to albumin binding and trafficking to cells [17,21].

Various foods serve as an exogenous source of AA for humans. Lean meat and meat fats, eggs, salmon and tuna contain high concentrations of AA [22,23,24], and AA is also derived from linoleic acid, an essential fatty acid, which is mainly found in vegetable oils and walnuts [25]. Endogenous AA generation mainly occurs via the release of AA from cell membrane phospholipids. This process is catalyzed by enzymes of the phospholipase A2 (PLA2) superfamily and is induced by various cellular activation signals, including inflammation or infection driven tumor necrosis factor receptor (TNFR) and toll-like receptor 4 (TLR4) stimulation [26,27]. Among the PLA2 enzymes superfamily, three members contribute to eicosanoid production and are involved in distinct functions of eicosanoid metabolism. The cytosolic Ca^2+^ dependent PLA2 (cPLA2) alpha mainly drives free fatty acids (FFAs) production and generation of AA, which is involved in cellular signaling. The cytosolic Ca^2+^ independent PLA2 (iPLA2) alpha contributes to cellular homeostasis via the synthesis of specialized pro-resolving mediators (SPMs) and reacylation of free AA, and the secretory PLA2 (sPLA2) controls free AA release and induces the local inflammatory response in a paracrine manner [27,28]. In addition to PLA2, two other phospholipase families, namely phospholipase C (PLC) and phospholipase D (PLD), generate AA via intermediate products such as diacylglycerol (DAG) [29,30]. Finally, endocannabinoids serve as an endogenous source for AA, as demonstrated by the generation of AA from anandamide [31].

**Figure 1:**
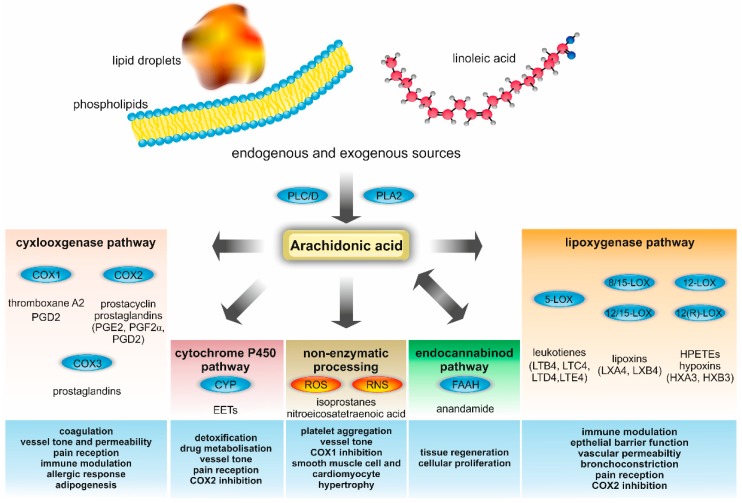
Metabolites and enzymes involved in AA metabolism and their biological functions in metabolic and cardiovascular diseases. Endogenous AA is mainly derived from cell membrane phospholipids, which are processed by phospholipase A2 (PLA2), phospholipase C (PLC), and phospholipase D (PLD). Free AA serves as a precursor for a plethora of metabolites, including prostaglandins (PGs), prostacyclin, thromboxane, HPETE, leukotrienes, lipoxins, hypoxins, anandamide, and epoxyeicosatrienoic acids (EETs). In addition to this enzymatic processing of AA, there is also a non-enzymatic metabolization. The latter is important for the production of isoprostanes and nitroeicosatetraenoic acid. Many AA metabolites are highly bioactive and involved in various crucial vital processes. Relevant biological functions in metabolic and cardiovascular diseases are summarized in the blue boxes at the bottom. Additional abbreviations used: COX, cyclooxygenase; CYP, cytochrome; ROS: Reactive oxygen species; RNS: Reactive nitrogen species; FAAH, fatty acid amide hydrolase; HX, hypoxin; LOX, lipoxygenase; LT, leukotriene; and LX, lipoxin.

Four enzymatic pathways process free AA, resulting in the generation of a plethora of AA derivatives with multitudinous functions (Figure 1). First, cyclooxygenase 1 (COX1) and 2 (COX2), also known as Prostaglandin G/H synthases, foster the production of thromboxane A2 (TXA2), prostacyclin (PGI2) and several prostaglandins (PGs) [32,33]. COX1 is constitutively expressed and found in all tissues, which are prone to lipopolysaccharide (LPS) induced inflammation. TXA2 and prostaglandins (PGs), such as prostaglandin D2 (PGD2), are metabolites of the COX1 pathway, and alter the vessel tone, mediate platelet aggregation and are implicated in immune surveillance [34,35,36,37,38,39]. COX2 is found in macrophages and endothelial cells, demonstrates high expression levels in the kidney and brain, and is induced by bacterial endotoxins, growth factors, hormones, and several cytokines [40]. Its major metabolites prostaglandin E2 (PGE2), PGI2, PGD2 and prostaglandin F2alpha (PGF2α) adapt host immune response, vessel tone regulation, thrombus formation, pain reception, and female fertility, and are involved in neurodegeneration and cancer [41,42,43,44,45,46,47]. Additionally, COX3, a splice variant of COX1, facilitates prostaglandin synthesis in the human brain and heart [48].

Second, four lipoxygenases (LOX), namely 5-LOX, 8-LOX, 12-LOX and 15-LOX, process AA. 5-LOX produces 5-hydroperoxyicosatetraenoic acid (5-HPETE), 5-hydroxyicosatetraenoic acid (5-HETE), and 5-oxo-eicosatetraenoic acid (5-oxo-ETE), as well as various leukotrienes (LTs) [49]. These metabolites affect neutrophil recruitment and diapedesis, epithelial barrier function, vascular permeability and bronchoconstriction [50,51,52,53,54]. 8-LOX and 15-LOX lipoxygenases convert free AA to 8- and 15-hydroperoxyicosatetraenoic acid (8-HPETE and 15-HPETE) and foster the production of 15-HPETE derivatives, such as 15-hydroxyicosatetraenoic acid (15-HETE), lipoxins and eoxins [55,56,57,58]. 12-LOX metabolize AA to 12-hydroperoxyeicosatetraenoic acid (12-HPETE), which itself serves as a precursor for 12-hydroxyeicosatetraenoic acid (12-HETE) and to hepoxilins [58]. AA derivatives from the 8-, 15-, and 12-LOX pathways are involved in the establishment of hyperalgesia and induce the expression of fatty acid translocase [59,60]. Lipoxins exert mainly anti-inflammatory properties. Interestingly, their synthesis is induced by the COX inhibitor aspirin, an antithrombotic agent widely used in the treatment and for secondary prevention of cardiovascular diseases [61,62].

Third, the cytochrome P450 (CYP450) pathway, which is mainly restricted to the liver and is well known for its function in detoxification, includes epoxygenase and ω-hydroxylase, which facilitate the production of epoxy-eicosatrienoic acids (EETs) and hydroxy-eicosatetraenoic acids (HETEs), respectively. These AA derivatives regulate vessel constriction and dilation, hamper hyperalgesia, and inhibit COX2 expression [63,64,65,66].

Fourth, anandamide, an endocannabinoid, is generated from AA via the fatty acid amide hydrolase (FAAH) in a reversible reaction [67], whereas the processing of anandamide to AA and ethanolamine takes place when tissue damage is associated with high levels of free AA [68]. In this situation, anandamide supports tissue regeneration, and cellular proliferation via interaction with cannabinoid type 1 (CB1) receptors [69].

Finally, free AA is also processed by non-enzymatic reactions. The four double bonds of AA are readily oxygenated to form bioactive molecules. Thus, oxidative stress and/or exposure of AA to reactive oxygen species (ROS) and reactive nitrogen species (RNS) result in oxidation of AA and generate isoprostanes and nitroeicosatetraenoic acids [70,71,72]. These eicosanoids were reported to inhibit COX1, whereas isoprostanes have been linked to platelet aggregation, vasoconstriction, smooth muscle cell proliferation, and cardiomyocyte hypertrophy [72,73,74]. To conclude, AA and its derivatives can enter numerous metabolic pathways that interconnect lipid metabolism with immunity. A synopsis of key enzymes, metabolites, and biological functions of the AA metabolism is depicted in Figure 1.

## 3. AA Metabolism in Human Health

In line with the plethora of AA derivatives and their functions, AA metabolism is important for human health and tissue homeostasis. AA itself confers cell membranes with flexibility and fluidity, serves as a lipid second messenger in cellular signaling, acts as an inflammatory intermediate and induces vasodilatation [27,75]. AA alters ion channel fluxes and voltage-gated proton pumps, including the modulation of Na^+^ channels in the heart, which are major contributors to cardiac excitability [76]. The modulation of proton pumps and pH is also essential for the NADPH oxidase mediated production of ROS by phagocytes, thus free AA induces oxidative stress [77,78]. As previously mentioned, oxidative stress per se has dramatic consequences on AA metabolism, as it alters AA release by PLA2, fosters auto-oxidation of AA and induces COX2 over-expression [79]. The induction of ROS by free AA is a major factor for its described roles in immune surveillance, apoptosis regulation and its possible tumoricidal activity [80,81], and is of special interest in cardiovascular and metabolic diseases, because oxidative stress is a relevant factor in the pathogenesis of DM, hypertension, hepatic steatosis, atherosclerosis, and heart disease [82]. In this context, oxidative stress induces insulin resistance in adipocytes and muscle cells via interaction with PI3-kinase and Akt signaling, decreases insulin secretion by β cells, alters cellular glucose uptake and lipogenesis [75]. Accordingly, antioxidants, such as NADPH oxidase inhibitors, are a promising treatment option for cardiovascular and metabolic diseases.

In humans, AA plasma levels typically range from 0.1 µM to 50 µM, whereas under pathophysiological conditions, AA plasma concentrations up to 500 µM can be found [83,84]. Interestingly, 50 µM to 100 µM of free AA are cytotoxic in vitro, thus it is likely that plasma AA concentrations found in the human body exert cytotoxic and apoptosis regulatory functions. Accordingly, AA is essential in embryogenesis and infant development, but may also be a driving factor in cardiovascular, metabolic and inflammatory diseases [85]. In line with this, several studies have found an inverse relationship of plasma AA concentrations and the risk of coronary heart disease [86,87,88]. Thus, it is important to elucidate any potential influence of AA on various aspects in human health.

AA metabolites are implicated in immune surveillance, the establishment of allergy, the initiation and resolution of an inflammatory response, platelet aggregation and the coagulation cascade, glucose metabolism, neuronal signaling, female fertility, and the adaptation of mood and appetite [17]. Interestingly, available enzyme cassettes to process AA and G-protein coupled receptors are different for each cell and tissue type, ensuring a cell/tissue-specific regulation and function of AA metabolism [89]. The functional state of cells (e.g., activated vs. non-activated macrophages) and tissue environment impact on the synthesis of AA derivatives, and the necessity for cell-cell interactions to produce specific eicosanoids, also termed transcellular biosynthesis, adds further complexity to AA metabolism [90,91]. Targeted lipidomic strategies using mass spectrometry-based technology platforms revealed that the compound effect of local lipid metabolites, and their time-dependent generation, determine the biological function of the AA metabolome, rather than the specific concentration of a single eicosanoid at a given site and time point [92]. This was also demonstrated by the partial inhibition of the immunomodulatory effects of FFAs by omega-3 PUFAs, such as eicosapentaenoic acid (EPA) and docosahexaenoic acid (DHA), which display anti-inflammatory properties and are precursors for pro-resolving SPMs [93,94]. SPMs, including resolvin D1, enhance phagosome formation in macrophages and are capable of reverssing the phagocytic defect of macrophages derived from diabetic mice. They may thereby counter-balance the effects of pro-inflammatory AA metabolites [95].

A detailed description of all functions of eicosanoids would go far beyond the scope of this review, but the interested reader may be referred to some excellent recent studies and reviews covering specific aspects of eicosanoid metabolism in more detail [13,16,33,64,92,96,97,98,99,100,101,102]. As far as the pathophysiology of metabolic and cardiovascular diseases is concerned, immune regulatory, pro-inflammatory, and inflammation resolving functions of the AA metabolism are of major importance [16,96,103]). Immune surveillance depends on the establishment of inflammation, which normally occurs as regulated and self-limited process. However, inflammatory stimuli may persist and mechanisms of resolution may fail, resulting in excessive or persistent inflammation in various pathophysiological conditions [99]. An acute inflammatory response is characterized by an initiation phase and a resolution phase and is clinically featured by the symptomatic complex of heat, swelling, pain, and eventually loss of function [96]. Both the initiation and resolution of inflammation are mainly controlled by local chemical autacoids, which present a potpourri of proteins, peptides and lipid-derived mediators (especially AA-derived leukotrienes and prostaglandins) [96]. These messengers regulate chemotaxis and function of leukocytes and alter the pro-resolving activity of tissue phagocytes. Whereas an acute inflammatory response is essential for host surveillance, dysregulation of pro-inflammatory and anti-inflammatory signaling cascades, as well as the lack of pro-resolving mediators, result in persistent tissue inflammation. The latter promotes tissue dysfunction, damage, and fibrosis, and has been implicated in various diseases [97]. Of note, ample evidence links chronic inflammation, even at low-grade levels, to the emergence and progression of metabolic and cardiovascular diseases [12,52,57,103,104,105,106,107], and various AA metabolites, especially prostaglandins, leukotrienes, and thromboxanes are highly bioactive and contribute to the establishment of unresolved chronic inflammation [16,97].

It has long been recognized that nutrition affects the functionality of immune and non-immune cells, and metabolic dysregulation results in a pro-inflammatory signaling in polymorphonuclear neutrophils (PMNs), macrophages, endothelial cells, and adipocytes [13,108,109]. Various diseases are linked to metabolic dyshomeostasis, including obesity, diabetes, NAFLD/NASH, and atherosclerosis [5,15,99,110,111]. The interaction of inflammation and metabolism is a double-edged sword: whereas metabolic stress induces inflammation, inflammation per se disrupts metabolic homeostasis. Thus, chronic metabolic or inflammatory diseases typically present a vicious feedforward cycle of dysregulated metabolism and inflammatory response.

Various trials have investigated the effects of alimentary PUFA supplementation and both omega-3 and omega-6 PUFA rich diets have been proposed to reduce the risk of cardiovascular and metabolic diseases. Consequently, dietary guidelines have included information on the appropriate consumption of omega-3 and omega-6 PUFAs, which aim to prevent deficiency of essential PUFAs and take possible beneficial and detrimental effects of alimentary PUFA supplementation into account. For instance, a FAO expert consultation report recommended a consumption of two-to-three percent of energy (%E) linoleic acid and 2.5 to 9 %E omega-6 PUFAs per day [112]. Still, these recommendations are controversial, because they are challenged by contrasting results of various trials and meta-analyses [113,114,115,116,117,118].

## 4. Alterations of Immunity and Wound Healing in Obesity and Metabolic Disease

Malnutrition, chronic over-feeding, and obesity are associated with the accumulation of triglycerides (TGs) in adipose tissue and ectopic TG deposition in various other tissues, including the skeletal muscle and the liver [15]. Lipolysis of TGs produces FFAs, which are highly toxic and induce pro-inflammatory signaling cascades in immune and non-immune cells via activation of pattern recognition receptors such as TLR2 and TLR4 [119]. Despite the accumulation of FFAs, obesity-associated exceedance of the body lipid storage and metabolization capacities also results in an overload with fatty acid intermediates, such as ceramides and DAG [120,121]. The latter are known precursors of AA thus foster the production of pro-inflammatory AA metabolites, including PGE2, leukotrienes, and HETEs, and an accumulation of DAG is implicated in insulin resistance [122,123,124]. The emergence of FFAs and induced eicosanoid pathways impair leukocyte function, contribute to PMN survival and persistence at inflamed sites, and decrease phagocytosis and efferocytosis, the local phagocytic clearance mechanism of apoptotic cells by tissue macrophages [5,125]. In contrast, anti-inflammatory AA metabolites, such as lipoxin A4 (LXA4), decrease obesity-induced adipose inflammation, attenuate pro-inflammatory M1 macrophages, and foster anti-inflammatory M2 macrophages [126].

In addition, nutrient excess is associated with chronic inflammation, and obese and diabetic patients are prone to infections [127]. This observation was mechanistically linked to an impairment of leukocyte function, as lipid exposure hampers macrophage phagocytosis and efferocytosis [128,129,130,131]. Interestingly, autocrine actions of COX2-derived prostaglandins, such as PGE2 and PGD2, are implicated in this process, linking AA metabolism to macrophage function and immune surveillance in obese and diabetic patients [125,132]. As previously noticed, AA and its metabolites affect cell membrane fluidity, which is also important for phagocytosis and containment of microbes and may thus represent another link between AA metabolism and macrophage function.

Prostaglandins are among the most potent pro-inflammatory mediators, as reflected by their contribution to the development of toxic shock syndrome [133,134]. Following LPS stimulation, the COX2 pathway elicits high levels of the severe fever mediator PGE2, and 5-LOX enzymes induce LT-mediated upregulation of adhesion molecules and accumulation of inflammatory leukocytes in affected tissues. Murine knockout models for COX2 and 5-LOX demonstrated that these eicosanoid pathways are essential in LPS-triggered fever and endotoxic shock [133,134]. On the contrary, murine infection models, introducing either intraperitoneal *E. coli*, intraperitoneal *Staphylococcus aureus* or *Staphylococcus aureus* skin infection, reported a beneficial effect of a co-treatment with antibiotics and eicosanoid-derived SPMs [93].

The susceptibility to infections and the immunomodulatory effects of lipid mediators are also implicated in impaired wound healing, another feature typical of obese and diabetic patients. Disturbed wound healing has been related to insufficient perfusion, neuropathy, micro- and macrovascular disturbances, and wound infections [135]. Leucocyte dysfunction and accumulation, as well as accumulation of apoptotic and necrotic cells along with defects in autophagy at sites of delayed wound healing, are pivotal contributors to this process and are triggered by the metabolic dysregulation in these individuals [130,136]. In this context, a study demonstrated that leptin receptor-deficient *db*/*db* mice, which present a genetic model for obesity and diabetes, display an impaired wound healing, which was associated with a blunted macrophage function and efferocytosis, and could be reversed by treatment with pro-resolving lipid metabolites [95]. Several AA metabolites, including PGE2, PGI2, leukotriene B4 (LTB4), leukotriene D4 (LTD4), and LXA4 are involved in wound healing [137]. Excessive LTB4 production has recently been linked to an increased risk for skin infection and impaired wound healing in diabetic mice, and was resolved following the blockade of LTB4 signaling [138]. In contrast, a beneficial effect of LXA4 containing microparticles in the topical treatment of skin lesions was described in a rat model [139] and PGE2 is involved in immune surveillance of the skin and supports dendritic and T helper (T_H_)-17 cell-mediated defense against staphylococcal skin infection [140].

## 5. AA Metabolism in the Pathogenesis of Diabetes Mellitus

Diabetes mellitus type 1 (DM1) and DM2 are by definition associated with recurrent hyperglycemia due to insufficient insulin production and insulin resistance, respectively. Hyperglycemia induces the production of pro-inflammatory mediators by PMNs, gives rise to oxygen radical formation, hampers PMN chemotaxis, and supports the adhesion of PMNs to the vasculature in diabetic mice [141]. Additionally, FFAs activate the NLRP3-ASC (for NACHT, LRR and pyrin domains-containing protein 3/apoptosis-associated Speck-like protein containing a CARD) inflammasome, and a disruption of the associated Nod-like receptors (NLRs) protects against insulin resistance and hyperglycemia in obesity [142]. Glucose and lipid metabolism share various metabolic pathways. Consequently, disturbances in glucose and lipid metabolism are tightly related, and over-nutrition and/or obesity ensue both, dysregulated lipid metabolism and hyperglycemia. Insulin sensitivity is determined by the interplay of multiple organ systems, namely pancreas, liver, skeletal muscle, adipose tissue, and the nervous system, whereas insulin production mainly depends on pancreatic β-cell function. High levels of FFA are accompanied by defective insulin signaling and β-cell dysfunction [119]. This effect of FFAs is mediated, at least in part, by TLR4/MyD88 (for Myeloid Differentiation primary response 88) signaling, and the recruitment of M1 macrophages to pancreatic islets [143]. Accordingly, TLR4-null mice are resistant to FFA induced adipose inflammation and insulin resistance [144]. Macrophages derived from diabetic mice have a pro-inflammatory phenotype and express high levels of acyl-CoA synthetase 1 (ACSL1). ACSL1 is implicated in the generation of pro-inflammatory prostaglandins, such as PGE2, thus fostering pro-inflammatory functions of macrophages [145]. Consequently, disruption of ACSL1 in myeloid cells significantly reduces the inflammatory signaling in diabetic macrophages and hampers the progression of atherosclerotic lesion in diabetic mice [145]. Similar to the previously described observations following FFA challenge, hyperglycemia in non-obese mice interferes with phagocyte function of macrophages, ensued by defects in bacterial clearance and efferocytosis [128,146]. Interestingly, AA metabolism is involved in both, FFA and hyperglycemia-mediated effects on macrophage function. AA is a strong inducer of insulin secretion, whereas its metabolites demonstrate a divergent contribution to insulin resistance, depending on the involved cells and tissues [147]. PGE2 hampers insulin secretion in pancreatic islets, and enhances pancreatic β-cell dysfunction and destruction, whereas PGI2 improves insulin sensitivity of pancreatic cells. Contrarily, PGE2 fosters adipogenesis in white fat tissue and induces glycogenolysis and gluconeogenesis, thus alleviating insulin resistance of adipocytes [147]. Recently, PGF2α, which is synthesized at higher levels in diabetic mice, was linked to hepatic gluconeogenesis, a major driver of fasting hyperglycemia in DM2 [148]. Additionally, the 12/15-LOX enzymes are linked to DM. 12/15-LOX enzymes induce the production of various HPETEs, which interact with peroxisome proliferator-activated receptor (PPAR)α and PPARβ, and are implicated in cytokine-mediated damage of pancreatic β-cells. 12/15-LOX knockout mice demonstrate a partial resistance to diabetes development [147,149,150,151]. Similarly, LTs, produced by 5-LOX and 12-LOX-derived HETEs, demonstrate inhibitory effects upon pancreatic insulin secretion, and genetic disruption or pharmacological inhibition of these LOXs protects against pancreatic islet cell destruction in diabetic mice [152]. In this context, LTB4 has been found to be essential for the recruitment and activation of adipose tissue B2 lymphocytes, which contribute to the establishment of insulin resistance following high-fat diet [153]. CYP450-derived EETs and 20-HETE induce insulin secretion and protect pancreatic islet cells from apoptosis [154,155]. Diabetes and obesity are associated with an enhanced expression of the soluble epoxide hydratase (sEH), a key enzyme in the degradation of EETs, and genetic deletion of sEH ensues an improved insulin sensitivity and an anti-apoptotic effect on pancreas islet cells in a murine diabetes model [156]. Accordingly, the therapeutic potential of sEH inhibitors was tested in several clinical trials. Whereas results of some trials are still pending (e.g., NCT03486223), a Phase II trial introducing a thrice-daily application of an orally administered sEH inhibitor in patients with mild to moderate arterial hypertension and pre-diabetes, failed to demonstrate an improvement of insulin sensitivity (NCT00847899). Finally, AA also facilitates the production of anti-inflammatory lipoxins. The latter were reported to improve insulin sensitivity and may prevent the development of DM [157]. For instance, LXA4 inhibits IL-6, TNF-α, and ROS production thus hampers obesity-associated inflammation and has an anti-diabetic effect [126,158,159,160]. Notably, a pooled analysis of twenty prospective trials investigating biomarkers for the eicosanoid pathway demonstrated a lower incidence of DM2 in individuals with higher proportions of linoleic acid biomarkers, suggesting that SPMs other than lipoxins harbor anti-diabetic features [161]. In summary, AA derivatives play diverse and partly contrasting roles in the pathogenesis of DM. Therefore, research in AA metabolism and its enzymatic pathways may identify novel targets for the treatment of DM and its associated co-morbidities.

## 6. AA Metabolites in Hepatic Steatosis and Steatohepatitis

The most prominent hepatic disease associated with mal- and/or excessive nutrition associated metabolic dysfunction is non-alcoholic fatty liver disease (NAFLD), which may progress to non-alcoholic steatohepatitis (NASH), hepatic fibrosis, and liver cirrhosis. NAFLD is the most frequent cause of chronic liver disease, with a reported worldwide prevalence of 25% [162], and is a main driver of increasing rates of hepatocellular carcinoma (HCC) in Western civilizations [163,164,165]. The prevalence of NAFLD closely correlates with an elevation of the BMI, dyslipidemia, DM, and insulin resistance [166]. An accumulation of intracellular lipids and the formation of lipid droplets (LDs) in the cytoplasm of hepatocytes without inflammation is the typical histological finding in isolated steatosis, whereas pathologic inflammation leading to cell necrosis is present in NASH. LDs were described to be a source of excessive production of pro-inflammatory eicosanoids, suggesting an early involvement of AA metabolites in NAFLD [167]. Progression from non-inflammatory steatosis to NASH is conceived as a sequential process, still pathophysiological features of isolated steatosis and NASH are quite distinctive [168]. Similar to other metabolic diseases, dysfunction of local tissue macrophages, namely Kupffer cells, and the activation of a lipid autocoid-cytokine-chemokine cascade ensued by the activation of stellate cells, are considered crucial factors in the development of the more progressive liver disease. Currently, a multiple hit theory is the most accepted concept to explain the pathogenesis of NAFLD and NASH [169]. In addition to environmental factors, such as fructose-rich nutrition and lack of exercise, genetic variants associated with alterations in lipid metabolism, hyperinsulinemia, insulin resistance, and immunometabolic effects of altered adipokine production (e.g., alterations in leptin, adiponectin, and resistin expression), changes of the gut microbiome, increased emergence of FFAs, and inflammatory lipid-mediators were found to contribute to disease progression [169,170,171,172].

The involvement of eicosanoids in NAFLD and NASH has been established by several lines of evidence. First, genetic disruption of PLA2, the rate-limiting key enzyme in AA production, alleviates high-fat diet-induced hepatic steatosis and results in reduced PGE2 levels in mice.

Second, 5-LOX-derived LTs are implicated in the pathogenesis of NAFLD, as both mice and humans suffering from NAFLD demonstrate a significantly increased activity of the 5-LOX pathway, and the enhanced expression of 5-LOX derivatives correlates with the severity of the hepatic disease [173,174]. A disruption of the 5-LOX pathway in hyperlipidemic apolipoprotein E (apoE) deficient mice reduces hepatic inflammation and tumor necrosis factor alpha (TNFα) dependent hepatic injury, and deficiency in leukotriene B4 receptor 1 (LTB4R, also known as BLT1), alleviates systemic insulin resistance in diet-induced obesity [175,176]. Accordingly, treatment with 5-LOX inhibitors mitigates hepatocellular steatosis and damage, and improves lipid transport via induction of very low-density lipoprotein (VLDL) secretion in leptin-deficient *ob*/*ob* mice. Similarly, genetic disruption of the 12/15-LOX pathway is protective against the development of NAFLD in hyperlipidemic mice [177]. To conclude, pro-inflammatory LOX pathways are considered major drivers of the progression of NAFLD to NASH, and the resolution of inflammation depends on the interaction of Kupffer cells and hepatocytes, to produce anti-inflammatory/pro-resolving lipoxins, such as LXA4 and lipoxin B4 (LXB4), via transcellular synthesis.

Third, Kupffer cells produce pro-inflammatory cytokines such as interleukin-6 and TNFα, as well as pro-inflammatory lipid mediators like the COX2-derived PGE2, which interact with hepatocyte lipid handling [178]. Interestingly, PGE2 promotes lipogenesis and inhibits tissue growth factor beta 1 (TGFβ1)-mediated collagen production by activated stellate cells, suggesting an anti-fibrotic function of PGE2 [179]. Additionally, the COX pathway is involved in the pathogenesis of NAFLD through its effects on PPARγ, which is implicated in insulin resistance and hepatic steatosis [180].

Fourth, enzymes of the CYP450 family are involved in both the production of anti-inflammatory EETs and HETEs [181], and the generation of ROS, which are considered to be a driving factor in steatohepatitis [182]. A role of CYP450 enzymes in NAFLD pathogenesis is also delineated by genomic analysis, demonstrating that several CYP gene variants determine susceptibility to the establishment of NAFLD/NASH [183]. Finally, serum levels of endocannabinoids are increased in obese and non-obese patients with NAFLD and may reflect an additional, thus far more poorly defined, role of eicosanoids in the pathogenesis of NAFLD [184].

In contrast, eicosanoids derived from omega-3 fatty acids exert a protective function against obesity-induced insulin resistance and NAFLD, as demonstrated by nutritional supplementation of omega-3 fatty acids in leptin receptor-deficient *db*/*db* mice. Moreover, in fat-1 mice, which display an increased omega-6 to omega-3 fatty acid ratio, transgenic expression of an omega-3 fatty acid desaturase restored omega-3 fatty acid levels, thereby critically improving steatohepatitis [185,186]. In line with this, omega-3 fatty acid-derived SPMs, such as resolvins, are protective against the development of NAFLD [96].

To date, no established pharmacological treatment is available for NAFLD and NASH. Still, changes in food intake, the use of anti-oxidant agents, anti-inflammatory lipid mediators, and disruption of lipoxygenase pathways offer promising treatment targets in NAFLD [170,174,175,176,187,188].

## 7. AA Metabolome in Atherosclerosis and Cardiovascular Disease

Atherosclerosis is characterized by a remodeling of arteries, leading to sub-endothelial accumulation of lipids and development of plaques, which narrow the arterial lumen and limit blood flow. Its cardiovascular complications, including heart failure and myocardial infarction, peripheral vascular disease, stroke, retinopathy, and kidney dysfunction contribute significantly to morbidity and mortality worldwide [2,11]. The pathophysiology of atherosclerosis resembles various features of metabolic and immunological changes found in obesity [189,190]. For instance, a significant role of lipids and inflammation is a striking feature of both diseases [1,8,11,13]. Hypercholesterolemia and lipoprotein imbalances, such as increases in low-density lipoprotein cholesterol (LDL-C) and enhanced production of oxidized LDL-C contribute to vascular wall inflammation via interaction with pattern recognition receptors of accumulating macrophages, as demonstrated for TLR4 and TLR2 receptors, and activation of these pathways are linked to progressive atherogenesis and increased cardiovascular risk [144,191,192]. Despite the historical view of atherosclerosis as a lipid storage disease, the involvement of pro-inflammatory monocytes and local tissue macrophages as major protagonists in the generation and progression of atherosclerotic lesions has expanded our understanding of its pathophysiology, and also provided mechanistic insight into the role of cholesterol in cardiovascular diseases [193,194,195,196]. Following chronic mal-/over-nutrition, macrophages in vessel walls engulf excessive amounts of oxidized LDL-C and transform into foam cells. These cells are known to have a pro-inflammatory phenotype and hampered phagocyte function. They critically contribute to the accumulation of cell debris and thrombotic material in atherosclerotic lesions. The release of pro-inflammatory cytokines by foam cells further increases the synthesis of atherogenic oxidized LDL-C, resulting in a vicious feed-forward loop of chronic local inflammation. Similarly, monocytes demonstrate a predominant pro-inflammatory phenotype in atherosclerosis. According to their heterogeneity in function and phenotype, different types of circulating monocytes, namely Ly6C^hi^ and Ly6C^low^ monocytes, have been identified in mice. Ly6C^hi^ monocytes have mainly pro-inflammatory functions, traffic to the atherosclerotic lesion and are considerably increased in hypercholesterolemic mice [193]. In contrast, Ly6C^low^ monocytes, which implicate in tissue homeostasis, are less prone to enter atherosclerotic plaques [197]. In humans, three subsets of monocyte populations have been characterized, namely CD14^++^CD16^−^, CD14^++^CD16^+^, and CD14^+^CD16^++^. In accordance with animal studies, the mainly pro-inflammatory CD14^++^CD16^+^ monocytes were associated with an increased risk of cardiovascular events and atherosclerotic plaque instability [198,199].

Hypercholesterolemia has been recognized since long as a central risk factor for the initiation and progression of atherosclerotic lesions [9], thus it is noteworthy that cholesterol and AA metabolism are interrelated. The latter was demonstrated by the inhibition of the 5-LOX pathway by the hydroxymethylglutaryl-CoA reductase antagonist atorvastatin, and the alteration of cholesterol homeostasis by the COX inhibitor aspirin. Specifically, aspirin induces the bile salt export pump Abcb11 and supports reverse cholesterol transport from atherosclerotic lesions to the liver for their subsequent biliary excretion [200,201]. Chronic hypercholesterolemia results in the sub-endothelial deposition of oxidized cholesterol-rich lipoproteins, which are involved in the recruitment and activation of macrophages, and generation of foam cells.

As previously mentioned, foam cells persist in the vessel wall and contribute to local inflammation and atherosclerotic lesion progression [107,110,111]. Additionally, PMNs foster plaque progression via pro-inflammatory functions and the production of reactive oxygen and nitrogen intermediates [105,202,203]. Interestingly, the PMN derived peptide S100A8 was found to orchestrate myelopoiesis and promote the recruitment of monocytes to atherosclerotic lesions [204]. AA metabolites are also implicated in this process as they contribute to chemotaxis, inflammation, and are involved in efferocytosis. Efferocytosis, especially the clearance of PMNs and foam cells, is of major importance for the resolution of inflammation, and its impairment leads to the progression of atherosclerotic lesions. Efferocytosis is actively supported by pro-resolving mediators, especially SPMs, which compromise various groups of eicosanoids: (1) Lipoxins produced by LOX enzymes, (2) E-resolvins derived from EPA, and (3) metabolites of DHA such as D-resolvins, protectins, and maresins [94,205]. In addition to their supportive role in efferocytosis, SPMs exert anti-inflammatory effects via inhibition of pro-inflammatory macrophage cytokine production and atheroprotective actions via induction of adhesion receptor expression in endothelial cells and reduction of intimal hyperplasia following vascular injury [206]. In this context, the AA derivative LXA4 is of special interest. LXA4 is a pro-resolving lipid mediator, which is mainly produced following the so-called “eicosanoid class switch”. This term was implemented to describe a switch in enzymatic AA processing, which is typically found in the resolution phase of inflammation [16,205]. During the early phase of inflammation, AA is predominantly metabolized via 5-LOX, which produces pro-inflammatory LTs including LTB4, whereas in the late phase PGs, such as PGE2, enhance 15-LOX expression, followed by a switch from LTB4 synthesis to 5-LOX and 15-LOX-mediated LXA4 production. Notably, in vivo LXA4 levels are decreased in patients with peripheral and coronary atherosclerosis [207], and overexpression of 12-LOX and 15-LOX in macrophages of atherosclerotic apoE deficient mice increases LXA4 production and hampers atherosclerotic lesion development [107]. This atheroprotective effect of LXA4 has been linked to its anti-inflammatory capacity, as it impairs the production of various pro-inflammatory cytokines, stops neutrophil chemotaxis, and induces pro-resolving macrophages functions [107,208,209]. Interestingly, aspirin enhances LXA4 production ensued by alleviation of atherosclerotic lesions in apoE deficient mice [210]. Notably, 12-LOX and 15-LOX are also key enzymes in the production of SPMs other than LXA4, thus the observed effects in this model may not be exclusively confined to LXA4. In addition, data on the atheroprotective functions of 12/15-LOX derived metabolites remains rather controversial, as earlier studies reported that 12/15-LOX- apoE- double-deficient mice (apoE^−/−^/12/15-LOX^−/−^) are less prone to atherogenesis, as compared to apoE-deficient littermates with fully functional 12/15-LOX [104].

In contrast to the mainly atheroprotective functions found for lipoxins, LTs affect the progression of hyperlipidemia-dependent vascular disease and are associated with atherogenesis, cardiovascular disease, myocardial infarction, and stroke [101,211]. LTs are AA metabolites derived from the 5-LOX pathway and exert their actions through binding to G-protein-coupled cell surface receptors. LTB4 interacts with leukotriene B4 receptor 1 and 2 (leukotriene B4 receptor 1 (LTB4R) and leukotriene B4 receptor 2 (LTB4R2), also known as BLT1 and BLT2), whereas cysteinyl leukotrienes (CysLTs), such as leukotriene C4 (LTC4), leukotriene D4 (LTD4), and leukotriene F4 (LTF4), bind to cysteinyl leukotriene receptor 1 and 2 (CYSLTR1 and CYSLTR2), respectively [212]. The CysLT leukotriene E4 (LTE4) displays a specific receptor, termed cysteinyl leukotriene receptor E (CYSLTRE) [213], and also interacts with the COX2 receptor PPARγ, and the purinergic receptor P2Y12 (P2RY12) [214,215]. LTB4 and CysLTs exert various effects on the cardiovascular system, including their negative inotropic action on the myocardium, the reduction of coronary perfusion via vessel contraction, the stimulation of arterial smooth muscle cell (SMC) proliferation and induction of the expression of adhesion and pro-coagulatory factors, such as von Willebrand factor (vWF), P-selectin and platelet-activating factor (PAF) [216,217,218,219,220]. Therefore, LTB4 and CysLTs are likely to contribute to the pathophysiology of atherosclerosis and myocardial dysfunction. Accordingly, an enhanced activity of the 5-LOX pathway was found in atherosclerotic lesions [221,222], and the quantity of 5-LOX positive cells correlates with the progression of atherosclerotic lesions and plaque stability [222,223]. Blockade of LTB4 receptors protects against the development of atherosclerosis in apoE-deficient mice [224], and the endothelial overexpression of CYSLTR2 increases vascular permeability, myocardial ischemia/reperfusion damage, and cardiomyocyte apoptosis in peri-infarct areas [54,225,226]. Notably, 5-LOX expression in atherosclerotic plaques is mainly restricted to local macrophages, and 5-LOX positive macrophages were implicated in the development of aortic aneurysms in apoE-deficient mice when kept on an atherogenic diet [211].

Genetic variations of key enzymes for LTB4 production, namely 5-LOX, 5-LOX activating protein (FLAP), and leukotriene A4 hydrolase (LTA4H), as well as alterations in their functionality are linked to atherosclerosis susceptibility and the prevalence of cardiovascular events [227,228,229,230,231,232]. Most recently, it was shown that LTB4 fosters the recruitment of neutrophils to atherosclerotic plaques and contributes to plaque destabilization [233]. In line with the pro-atherogenic effects of LTs, they are implicated in myocardial ischemia and reperfusion injury. Accordingly, endothelial cysteinyl leukotriene 2 receptor (CYSLTR2) expression within the heart and vasculature is induced by ischemia/reperfusion injury [234]. Interaction of LTs with CYSLTR2 increases vascular permeability and amplifies the extent of the myocardial injury, and high levels of CYSLTR2 expression in the heart and vessels have been linked to a detrimental outcome in murine ischemia/reperfusion models [234]. In line with this, pharmacological blockade of LTBR4 reduces infarct size in a murine model of myocardial ischemia/reperfusion injury [235], and the CYSLTR antagonist montelukast, which is mainly used in the treatment of asthma and allergic rhinitis, was recently evaluated for its possible cardio-protective capacities. Interestingly, both animal models and clinical trials demonstrated a preventive role of montelukast against the development of atherosclerosis and suggested a cardioprotective function [236,237,238,239,240].

The COX pathway is one of the major treatment targets in atherosclerotic and ischemic heart disease because it affects major pathophysiological features of these diseases, including platelet aggregation, vessel wall tension, and inflammatory processes in atherosclerotic lesions [102]. The anti-inflammatory and anti-thrombotic features of aspirin, the only known irreversible inhibitor of platelet COX1, are primarily related to the suppression of PG and TXA2 synthesis [241]. In this context, a recent study demonstrated that the COX1/TXA2 pathway is a relevant contributor to vascular hypercontractility in atherosclerotic apoE deficient mice, and its pharmacological inhibition improves endothelial function [242]. Despite being a well-established therapy in cardiovascular disease, some aspects of aspirin treatment warrant further investigation, as reflected by a recent analysis demonstrating an interaction of body weight with the effectiveness of aspirin to prevent cardiovascular events [243]. Notably, aspirin is the only known non-steroidal anti-inflammatory drug (NSAID) with cardioprotective effects. On the contrary, NSAIDs with COX2 inhibitory features increase the cardiovascular risk, which is mainly attributed to their blockage of prostacyclin production [244,245]. Recently, a new drug class, which blocks both the COX1/2 and 5-LOX pathway in a balanced fashion, is evaluated in clinical trials and may present and additional treatment option for cancer, inflammatory, and cardiovascular diseases [246].

The hepatic CYP450 enzyme family is also linked to the pathophysiology of atherosclerosis [66]. The CYP450 enzymes have a well-established function in the regulation of systemic blood pressure, as EETs exert vasodilatory effects, and are implicated in cardiovascular and renal disease [66,247]. Accordingly, the pharmacological inhibition of the soluble epoxide hydrolase (sEH), a key enzyme in the dissipation of EETs, exhibits antihypertensive and cardioprotective activity in transgenic rats with angiotensin II-dependent hypertension [248]. As previously mentioned, oral administrations of sEH inhibitors are currently tested in clinical trials and may present a future therapy in patients with cardiovascular diseases and arterial hypertension [249].

Finally, AA metabolites derived from non-enzymatic AA processing, such as isoprostanes may be involved in the pathophysiology of cardiac diseases. For instance, 15-F2c isoprostane activates the PGF2α receptor and induces hypertrophy in cardiac smooth muscle cells [73]. Still, data on isoprostanes in atherosclerosis and cardiac disease are sparse and urge further elucidation.

## 8. Conclusions and Perspectives

Metabolic and cardiovascular diseases share the common pathophysiology of unresolving chronic inflammation. AA metabolites are important factors in the initiation and resolution of inflammation and have been linked to the pathophysiology of obesity, DM, NAFLD/NASH, and cardiovascular diseases. The plethora of AA derivatives and their multitudinous implications, and the differential expression and functionality of AA metabolites in various cells and tissues, as well as their transcellular biosynthesis, render the AA metabolism one of the most complex regulatory systems within the human body. It is thus not surprising that many aspects of the AA metabolome still need further investigation, despite extensive scientific progress in the field. The establishment of new methodologies, including real-time and cell-specific lipidomic profiling, commence an opportunity to achieve a better understanding of the complexity of the eicosanoid metabolism and may help to improve current treatment strategies and to establish new approaches to counteract metabolic and cardiovascular diseases.

## 9. Methodology

We collected data and references with PubMed (available online: https://www.ncbi.nlm.nih.gov/pubmed/), Google, and Google Scholar. The following keywords were used for literature search: arachidonic acid, eicosanoids, isoprostanes, cholesterol, phospholipases, cyclooxygenases, leukotrienes, lipoxygenases, CYP450, epoxygenases, obesity, metabolic disease, NAFLD, cardiovascular disease, atherosclerosis, and DM.

## Abbreviations

%E	Percent of energy
AA	Arachidonic acid
ACSL1	Acyl-CoA synthetase 1
ALOX; LOX	Lipoxygenase
apoE	Apolipoprotein E
*BMI*	Body mass index
*CB1*	Cannabinoid type 1 receptor
*COX*	Cyclooxygenase
*CYP450*	Cytochrome P450
CysLTs	Cysteinyl leukotrienes (LTC4, LTD4, LTE4 and LTF4)
CYSLTR	Cysteinyl leukotriene receptor
*DAG*	Diacylglycerol
DHA	Docosahexaenoic acid
DM	Diabetes mellitus
EET	Epoxyeicosatrienoic acid
EPA	Eicosapentaenoic acid
FAAH	Fatty acid amide hydrolase
FFA	Free fatty acid
HETE	Hydroxyeicosatetraenoic acid
HPETE	Hydroperoxyeicosatetraenoic acid
HHT	12-Hydroxyheptadecatrienoic acid
LDL	Low density lipoprotein
LDL-C	Low density lipoprotein cholesterol
LPS	Lipopolysaccharide
LT	Leukotriene
LTB4	Leukotriene B4
LTB4R	Leukotriene B4 receptor
LXA4	Lipoxin A4
MyD88	Myeloid differentiation primary response 88
NAFLD	Nonalcoholic fatty liver disease
NASH	Nonalcoholic steatohepatitis
NLRP3-ASC	NACHT, LRR and PYD domains-containing protein 3/apoptosis-associated Speck-like protein containing a CARD
NLR	Nod-like receptor
oxLDL-C	Oxidized low density lipoprotein cholesterol
P2RY12	Purinergic receptor P2Y12
PAF	Platelet-activating factor
PG	Prostaglandin
PGI2	Prostacyclin
PLA_2_	Phospholipase A2
PLC	Phospholipase C
PLD	Phospholipase D
PMN	Polymorphonuclear leukocyte
PPAR	Peroxisome proliferator activated receptor gamma
PUFA	Polyunsaturated omega fatty acid
RNS	Reactive nitrogen species
ROS	Reactive oxygen species
sEH	Soluble epoxide hydratase
SMC	Smooth muscle cell
SPM	Specialized pro-resolving mediators
TG	Triglyceride
T_H_	T helper cell
TLR	Toll-like receptor
TNFα	Tumor necrosis factor alpha
TXA2	Thromboxane A2
VLDL	Very low-density lipoprotein
vWF	Von Willebrand factor
WHO	World Health Organization
